# Observations on the Employment of Compression in Aneurism

**Published:** 1847-03

**Authors:** O’Bryen Bellingham

**Affiliations:** The Medical Officers of St. Vincent’s Hospital


### RECORD OF MEDICAL SCIENCE.

*Observations on the Employment of Compression in Aneurism.* By O'Bryen Bellingham, M. D., F. R. C. S. I., one of the Medical Officers of St. Vincent's Hospital.

### *Mode in which Nature effects the cure of Aneurism.*

When we consider how many writers have devoted their attention almost exclusively to the subject of aneurism, and how much talent has been engaged in illustrating its history, pathology, and treatment, it appears strange that the process which nature herself sets up for its cure should have been so much overlooked hitherto by surgeons; and although this process was daily, I may say, passing under their eyes, that the exact mode in which it was accomplished should have attracted but little attention, and no attempts should have been made to imitate or assist it.

In almost every case of aneurism where the disease has subsisted for some time, we find a larger or smaller amount of solid matter deposited in the sac, which is composed of the fibrine of the blood, arranged in regular concentric laminæ.

Examples of the spontaneous cure of aneurism, in which the sac is completely filled with fibrine deposited in regular concentric layers, are not very uncommon.

Again, in cases of valvular or other disease of the heart, when a considerable impediment exists to the circulation through its chambers, we know that the fibrine of the blood will separate from its other constituents, and form the bodies improperly termed polypi, (which are sometimes so closely interwoven with the carnæ columnæ and chordæ tendinæ as to be with difficulty detached,) which by closing the orifices, or obstructing the action of the valves, not unfrequently proves the immediate cause of death.

These familiar facts all tend to prove—

1st. That nature herself sets up a process by which, under favourable circumstances, the cure of aneurism is effected.

2d. That the mode in which she effects this, always in internal aneurism, and frequently in external aneurism, is by the deposition of the fibrine from the blood in the sac of the aneurism, until it becomes filled.

3d. That the fibrine in such cases is deposited in regular concentric laminæ, the oldest or first formed next the sac, those most recently formed nearest the centre.

4th. That a current of blood through the aneurismal sac is a necessary agent in bringing about this result.

5th. That any obstruction to the current by which its velocity and amount are diminished will accelerate the deposition of fibrine in the aneurismal sac.

6th. That once this process has commenced, if the same agents continue in operation, it will go on until the sac becomes filled, and no longer permits of the entrance of blood.

Writers upon aneurism hitherto appear to have been more intent upon solving unimportant points connected with the distinction between true and false aneurism; or in ingenious speculations as to the comparative frequency of aneurism from dilatation of all the coats of the artery, or from rupture of the internal and middle coats, than in investigating the mode in which a spontaneous cure of the disease takes place. Indeed so little notice is taken of this process in some modern works, that one would suppose the authors were either ignorant of the facts just stated, or looked upon the phenomena as too unimportant to dwell upon.

Before proceeding further, there is a point upon which I wish to make a few remarks. In the details of the cases of aneurism treated by compression which have been published within the last three years, the writers speak of the coagulation of the contents of an aneurismal sac, or of developing a coagulum in it by pressure upon the artery at the cardiac side, as if a coagulum or clot, and the concentric laminæ of fibrine which form in aneurisms, were identical: indeed, from the loose manner of expression adopted, it is sometimes difficult to tell whether the writers are aware of the distinction between them. This is the more remarkable, because the two substances in appearance, colour, and consistence, present a remarkable contrast; the one being soft and loose, of a very dark colour, not deposited in any regular order, and composed of the red globules and fibrine of the blood; the other being solid and firm, of a paler colour, deposited in regular concentric laminæ, and composed of fibrine alone, or with a very small proportion of the red globules. The former is commonly found in the auricles of the heart, and in the large veins which open into them, and is familiar to every body; the latter constitutes the solid matter, which, in greater or less quantity, fills the sac of old aneurisms.

It is obvious, therefore, that the mode in which these two different deposits are formed cannot be the same. To cause the deposition of fibrine in an aneurismal sac, it is essential that a stream of blood should pass through it for a period that will vary according to different circumstances; but its deposition will be promoted or encouraged by diminishing the strength of the current in the artery leading to it, and by lessening the amount of blood which passes through the sac. This, it is easy to understand, can be readily accomplished by compressing the artery at the cardiac side of the aneurism, and the pressure need not be so strong as to occasion very great pain to the patient.

On the other hand, to bring about the coagulation of the contents of an aneurismal sac, the blood must remain at perfect rest for a considerable time; if a current continues to pass through the sac, the blood in it will be replaced by another portion before there is time for its coagulation; for although a coagulum or clot will quickly form when blood is removed from a vein, it is not so easy a matter to bring about its coagulation in an aneurismal sac in a living subject. To effect this, very considerable pressure would be necessary; the compression likewise, it appears to me, would require to be made upon both the cardiac and capillary side of the sac, and very near the latter; while the process will necessarily be so painful that few patients would be willing to submit to it.

It would appear then—

1st. That it is not by the formation of a coagulum in the aneurismal sac that nature effects the cure of the disease, but by the deposition of the fibrine from the blood which circulates through the sac.

2d. That simply diminishing the current will not cause the coagulation of the contents of an aneurism, but it will occasion the deposition of fibrine in the sac.

### *Proofs that Compression effects the cure of Aneurism in the same way as nature accomplishes this object.*

The details of the cases previously given prove that pressure, so as completely to prevent the entrance of blood into an aneurismal sac (supposing that this could effect the coagulation of the blood, or that it could be borne by the patient,) is not necessary for the cure of aneurism; and all the facts connected with this method of treating aneurism go to prove that the consolidation of the tumour is brought about by the deposition of fibrine in the sac, not by the coagulation of the blood contained in it. Thus it has frequently been mentioned that at the commencement of the treatment, the aneurismal tumour was soft and compressible, and collapsed on pressure on the artery leading to it; but after compression had been employed for a time, the sac became hard and incompressible, and did not collapse when the circulation through the artery leading to it was interrupted; proving that a deposition of fibrine had taken place, and that the process by which the sac was to be filled up had commenced.

In two cases recently published, an abstract of which has already been given, (one of which was treated by Mr. Porter, and the other by Mr. Cusack, and where the patients, curiously enough, were both medical men,) it has been mentioned that after pressure had been employed for a time, it became so irksome that the patients refused to continue it, they resumed their ordinary habits, and commenced taking exercise. At this period the sac in both cases is stated to have become hard and incompressible, yet the aneurisms ceased to pulsate, and a cure was effected, although pressure had not been used for some time; showing that the deposition of fibrine having commenced, went on, notwithstanding that compression was discontinued.

Again, I have given an account of the appearances found in the limb of a patient the subject of popliteal aneurism, who had been under treatment by compression for some time, but who died of erysipelas before the cessation of pulsation in the aneurism, and I have stated that "the aneurismal sac was in a great measure filled with firm layers of fibrine deposited in concentric laminæ," but that "no coagulum or clot was contained in the sac."

Lastly, the very fact of the irregular manner in which the compression has been maintained in many of the cases, having been often intermitted and resumed according to the sensibility or irritability of the patient, is an additional proof that it was by the deposition of fibrine in the sac, not by the formation of a coagulum, that the cure was ultimately accomplished.*

From what I have now said, it would appear that the mode in which pressure upon the artery at the cardiac side of the aneurism effects the consolidation of an aneurism, and that by which nature effects this object, are identical; in both cases the fibrine of the blood is

*I have often been compelled, much against my will, to digress, in order to correct the misrepresentations and errors contained in a "History of the Cure of Popliteal Aneurism by Compression," put forward in the shape of an "editorial article" by Mr. Wilde, in the *Dublin Quarterly Journal of Medicine* for August, 1846 (which has been also reprinted separately, and distributed extensively.) In noticing this curious production for I hope the last time, I shall merely observe, that his remarks upon the mode in which compression effects the cure of aneurism will be read with surprise by every one who has seen this method employed; and cannot fail of leading to the conclusion that the writer must be utterly ignorant respecting both its theory and practice. "It is quite apparent (he says, the italics are also his own.) that its [pressure] removal at *a particular time*, even for a few minutes, and allowing the flow of blood through the sac again to take place, will undo all that had been before effected!"—*Vide Dub. Quart. Jour. for Aug.* 1846, *p.* 131.

gradually deposited in the sac, which continues until the sac becomes filled, and no longer permits of the entrance of blood. I have next to show that the ulterior changes which take place are the same in both.

When the sac of an aneurism has been completely filled by fibrine, deposited as already mentioned, the disease is cured; it can neither increase in size, nor can rupture take place. If the aneurism was seated in the arch of the aorta, however, the calibre of the artery at the part from which it springs will still be preserved, and the circulation will continue through it as before, because the anastomosing branches are not sufficiently numerous or large here to carry on the circulation if this vessel was suddenly obliterated; while the current through it is necessarily too strong to permit of fibrine being deposited in its interior. Eventually, the sac diminishes in size from the gradual absorption of its contents, and ultimately it may in a great mea sure disappear.

When the sac of an external aneurism has become filled by fibrine, the disease is evidently also cured, and the circulation through the artery may continue for a time; but the anastomosing branches here being numerous and free, the blood passes down the main artery of the limb in a diminished stream, the deposition of fibrine therefore continues until the artery at the seat of the aneurism is encroached upon and gradually filled by fibrine, when its pulsation stops, and the deposition of fibrine ceases.

Once a certain amount of fibrine has been deposited in an aneurismal sac, as the result of pressure upon the artery above, there would appear to be an irresistible tendency to its continuing to be deposited until the artery at the part is likewise closed up; this fact has been proved by the two cases to which I have recently referred, where the patients refused to continue the pressure, and commenced taking exercise; yet under these apparently unfavourable circumstances, the pulsation ceased after a short interval, and a cure was effected. It is therefore not unlikely that the artery has many times been tied in aneurism, where the process which nature sets up for its cure had made considerable progress, and where a little further delay would probably have done away with the necessity for its performance.

The ultimate changes which take place when the pulsation of an external aneurism ceases, are the same, whether this result has occurred spontaneously or been brought about by compression. The circulation is carried on by the enlarged collateral vessels, the contents of the sac and the artery at the part from which it springs, are gradually removed by the absorbents, the sac disappears in a great measure, and the vessel is eventually converted into an impervious ligamentous band. Guattani Petit, besides many other writers, have reported cases of aneurism spontaneously cured, where this condition of the parts was found; and I have already described the appearance which I found in a case were the patient died two years and a half after the cure of a popliteal aneurism by compression, and sixteen months after the cure of a femoral aneurism in the opposite limb;

"the artery at the site of each anurismal sac was converted into a solid, thick, ligamentous band, its channel was obliterated, and the contents of the sacs had been removed by the absorbents."

The foregoing facts all go to prove, that when a cure of external aneurism takes place spontaneously, or when it is brought about by compression, it cannot fail of being permanent; the sac is filled up, and the artery from which it springs is obliterated; consequently, pulsation cannot return, nor can a secondary aneurism form at the part, both of which have occasionally occurred after an apparent cure by the ligature.

*Proofs that the ligature at a distance from the sac effects the cure of aneurism in the same way as compression.*

I have now given a sufficient number of proofs that the mode in which compression effects the cure of aneurism, is identical with that by which nature accomplishes this object. If it could now be shown that the ligature of the artery at a distance from the sac generally effects the cure of aneurism in the same way, it might help to remove the prejudiced view taken of compression by surgeons, who are either so wedded to the ligature that they distrust every other mode of treating aneurism; or who, through ignorance of the principles upon which it effects a cure, can see in it nothing but the revival of an obsolete and abandoned method. This I shall now endeavour to do.

Almost every writer upon aneurism mentions cases where the pulsation returned after the ligature had been used; or where hæmorrhage occurred from the sac subsequent to the operation. "It is not uncommon (Mr. Guthrie says) for a pulsation to be felt in the tumour a few hours, or in a day or two after the operation, but it very rarely continues." "A stream of blood (Mr. Hodgson observes) in most instances passes through the aneurism after the ligature of the superior part of the artery; yet this current is in most instances not sufficient, either by its quantity or by the force with which it is impelled, to continue the disease."

These facts show that the ligature of the artery at a distance from the aneurism does not always prevent a current of blood from passing through the sac, while pathology proves that inosculating branches not unfrequently communicate with the artery between the ligature and the sac; that the artery is always pervious here, and remains so after the cure of the aneurism; and that the sac has been found filled with fibrine deposited in concentric layers, where an opportunity has been afforded for examining the limb some time after the operation.

The theory laid down in modern works respecting the mode in which the ligature of the artery at a distance from the sac effects the cure of aneurism, is as follows:—When the ligature is tightened, the blood contained in the sac, or which may find its way into it afterwards, distends the sac, and extends into the artery above and below the opening by which it communicates with it; it coagulates, the fluid parts are absorbed, and the more solid, aided by the contraction of the sac, form a firm tumour. The coagulum is subsequently removed by the absorbents, and the vessel is obliterated at the seat of the aneurism, and for a short distance above and below it. The sac gradually diminishes in size from the absorption of its contents, and ultimately disappears either wholly or partially.

The foregoing theory would be perfectly correct if the artery were always tied close to the aneurismal sac, or if the artery were always obliterated between the ligature and the sac. But as the ligature is applied at a distance from the sac, and as the vessel in nineteen cases out of twenty remains pervious afterwards for some space between the ligature and the sac, it is evident that the vessel here must have continued to convey blood after the operation, because otherwise its cavity would eventually have been obliterated. It is therefore probable that a feeble current of blood continues to pass through the sac after the operation, and that in a great majority of cases the cure is brought about by the deposition of fibrine in the sac, not by the coagulation of its contents. This is further confirmed by pathology; Mr. Hodgson says, "in the dissection of cases some time after the operation, it has been found that the cavity of the sac was filled with concentric layers of coagulum, similar to that which is met with in aneurisms undergoing spontaneous cure," which obviously could not have occurred unless a current of blood continued to pass through the sac after the operation.

The ligature of the artery at a distance from the sac cannot of course effect the cure of aneurism unless the sac itself becomes obliterated: now, it appears to me that if the theory usually laid down were correct—viz., that the blood contained in the sac, and that which finds its way into it subsequently, coagulates after the ligature is tightened, one of two results would follow the operation much more frequently—a secondary aneurism would form at the part: or suppuration of the sac would ensue.

A secondary aneurism, for instance, would be liable to form, if the sac was large, and its contents wholly fluid at the time of the operation. It is obvious that in such a case, when the fluid portion of the coagulum is absorbed, the sac must shrink considerably to form a solid tumour. Writers upon aneurism have, however, endowed aneurismal sacs with extraordinary elastic powers, in order to enable them to uphold this theory; but in all the cases which I have seen dissected, the sac had formed adhesions with the parts in its vicinity; and if it possessed elasticity (which, considering that it is composed of the cellular coat of the artery is rather doubtful,) this agent could not have come into operation; consequently if a large anastomosing branch eventually communicated with the artery between the ligature and the sac, the pulsation would return, and a secondary aneurism would form at the part. I have little doubt that in the cases which have been reported where this result followed the operation, it was owing to a coagulum only having formed in the sac; it evidently could not have occurred if the sac was filled by fibrine deposited in the way that had been mentioned.

That suppuration of the sac would be liable to ensue under such circumstances, is, I think, also evident. We have a large sac containing more or less coagulated blood, this after a time comes to act as a foreign body, inflammation followed by suppuration sets in, particularly if, as not unfrequently happens in hospital practice, the tumour is much or often handled. The history of operative surgery contains many cases where suppuration of the sac followed the operation; and what has now been said will explain why it should have occurred in some cases and not in others apparently similar. If the ligature is placed very close to the sac, and the current of blood is thus completely cut off from it, inflammation followed by suppuration is more liable to follow than where the vessel is tied at a distance from the sac; we have seen that in the latter case a feeble current continues to pass through the sac after the operation, the sac becomes eventually filled with fibrine, and inflammation is as unlikely to ensue as after the treatment by compression.

Writers upon aneurism are somewhat obscure as to the exact mode in which the *artery* at the seat of an aneurism is obliterated after the ligature. The examination of cases where the patient lived for a long time after the operation, proved that this did occur; and that the appearances found here on dissection were exactly similar to those observed after both a spontaneous cure and a cure by compression.

The explanation usually given is, that the coagulum extends from the sac into the artery above and below it, which prevents the entrance of blood; this after a time becomes absorbed, the artery at the part gradually contracts, and is eventually converted into a solid cord. But we have seen that the artery remains pervious for some distance between the point at which the ligature is applied and the sac, and that a current of blood probably continues to pass through it after the operation, which would necessarily interfere with the formation of a coagulum in the artery at this part. Others suppose that inflammation sets in here, which unites the opposite sides of the artery; but they do not give any reason why the adhesive inflammation should be so conveniently set up at this particular spot; besides, we know that the vessel is obliterated at the same place when a spontaneous cure occurs, or where this followed the employment of pressure, although no adhesive inflammation occurs in either of the latter cases. The explanation becomes easy, however, if we admit that a current of blood continues to pass through the sac after the operation; the oblitration of the artery will be effected exactly in the same way as occurs in cases of spontaneous cure, or where compression had been employed.

Every thing that has now been said makes it probable that the ligature of the artery at a distance from the sac, and compression of the vessel at the cardiac side, effect the cure of aneurism in the same way. It has already been shown that the mode in which nature brings about a spontaneous cure is precisely similar; which of itself is a strong argument in favour of its correctness. But there is another circumstance which tends still further to confirm it—viz., that several phenomena connected with the surgical treatment of aneurism which were hitherto obscure, and inexplicable upon the theory that the coagulation of the contents of the sac always follows the operation by ligature, are readily explained according to this theory.

For instance, we have seen that a slight pulsation is not unfrequently felt in the sac subsequent to the operation. Now, this theory not only explains its cause, but it shows why it ceases after a short time, and why, instead of being an unfavourable sign, it should rather be regarded as a favourable one.

By this theory alone, we can account for the artery being obliterated after the ligature at a point from which the aneurism springs, while it remains pervious between the ligature and the sac.

Lastly, it enables us to explain why suppuration of the sac should occur after the operation in one case and not in others apparently similar; and how a secondary aneurism forms in certain cases a long time after the operation, while in general it must be a rare result of the ligature.

*Spontaneous cure of external Aneurism.*

Every writer upon aneurism mentions or refers to cases where an external aneurism ceased to pulsate some time after admission into hospital, although a bandage had been merely placed round the limb (with or without compress upon the tumour,) but without the slightest intention or expectation of producing any effect upon it. Such cases are comparatively rare, and hitherto have been looked upon as the result of some happy chance which might occur once or twice in a practitioner's life, but from which no deductions could be drawn. "Such instances of inexplicable recovery (a recent writer observes) are extremely rare, and as examples of singular good fortune, are rather to be hoped for than expected; neither can any principle of practice be established on them."

In Mr. Porter's excellent practical treatise on Aneurism, published a few years since, two cases of this kind, which occurred in the Meath Hospital, are shortly reported. "Some years since (he says) a man suffering from aneurism was admitted into the Meath Hospital. The tumour was situated low down in the popliteal space, and was large, being fully the size of a turkey's egg. The limb was semiflexed, and could not be extended; pain very considerable; tumour not compressible, at least pressure influenced its size but slightly; it was hard, and did not diminish in bulk when the femoral artery was compressed, which, however, stopped the pulsation. With a view to humour the patient, until he could be persuaded to submit to an operation which I conceived to be absolutely necessary, I rolled a large bandage round the entire limb, from the toes upwards. This, as the idea of treating the disease by compression had never been contemplated, was very loose; nor had I the least notion that the tumour could have been influenced by it, one way or another. But on my visit the next day the aneurism was gone. Within an hour after the application of the bandage, the patient experienced some pain in the tumour, which soon became excruciating, and continued during the entire night. In the morning the tumour no longer pulsated—it had become solid and firm, and eventually the disease was cured."

"On another occasion a man was admitted into the same hospital, under the care of Mr. M. Collis, with a popliteal aneurism, the history of which I do not recollect with very great accuracy, except that it was rather of large size, not compressible, and there seemed to be not much fluid blood in the sac. A bandage was applied to this in a similar manner to that in the former case; it caused immense pain, and on the following morning the pulsation in the tumour was no longer to be felt. It, however, reappeared after a little time, but so very indistinctly that it was a questionable matter whether the sensation was not communicated from the finger of the examiner, and not from the tumour. The application of the bandage was persevered in, and in the course of a very few days no doubt could be entertained of the cure."

In the second volume of the Dublin Medical Press, Dr. Brunker of Dundalk, has related a somewhat similar case. The patient, aged 32, was admitted into the Louth Infirmary in August, 1839. His occupation had been very laborious and the aneurism had been first observed fifteen months previously. "On examining the ham, the entire space was observed to be completely filled up by a pulsating tumour, beating synchronously with the heart, soft and compressible, but becoming distended on the removal of the pressure. Pressure being made on the femoral artery in the groin, the pulsation instantly ceased, and the size of the tumour was sensibly diminished. He was ordered to remain in bed, and was placed on low diet.

"Two days after admission, a piece of dry sponge was placed over the aneurism, and retained *in situ* by a roller rather loosely applied."

Five days subsequently, on the removal of the bandage and compress, the tumour was much diminished in size, and no pulsation whatever could be detected in it. The pressure was continued, and the patient was directed to remain in the recumbent posture. He left the infirmary a few days afterwards. He was seen about a fortnight subsequently, and the bandage and compress had been continued, and he had remained quiet. "On removing them (Dr. Brunker observes) I could scarcely discover any traces of the former tumour, and no pulsation whatever was to be felt. A very obscure pulsation was to be felt in the anterior tibial artery, which could not before be discovered on a very careful examination."

It appears to me that the cause of the cessation of pulsation in the cases of aneurism which have been detailed, and in other similar ones, instead of being obscure, is easily explicable upon the principles laid down in the preceding pages; aud that their history tends still further to corroborate what I have already said respecting the mode in which compression effects the cure of aneurism.

Thus, in every case that has been reported, the disease was of long standing, and the tumour had attained a considerable size. In the majority of cases, likewise, it was hard and solid, and did not diminish in size when the artery above was compressed; proving that a deposit of fibrine had already taken place in the sac, and that nature had already made some progress in the process by which the cure was ultimately to be accomplished. In fact, the condition of the parts was similar, in almost every respect, to that which we endeavour to bring about by the application of pressure; and which when it occurs, is the most satisfactory proof that the case is progressing towards a cure. In the third case which I have quoted, the tumour, though of long standing, is reported to have been "soft and compressible;" but it will be observed that pressure was maintained for five days before the aneurism ceased to pulsate, evidently a sufficient length of time for a deposit of fibrine to take place. Indeed in some of the cases of aneurism treated by compression, previously given, the pulsation ceased within a shorter period.

The mode in which the bandage and compress acted in effecting the cure of the aneurism in these cases appears to be similar to that in which nature in some rare instances, has effected this object—viz., the solid, or nearly solid sac was pressed against the artery from which it sprung, or against the orifice by which the sac communicated with the artery, in consequence of which the stream of blood which entered the sac was much diminished in amount and force, the disposition of fibrine then went on rapidly until the sac and artery at the part were obstructed; when, of course, the pulsation ceased.

Although "no principle of practice could be established" upon such cases, as long as the old theory respecting the cure of aneurism continued to be entertained, yet now that it has been proved that the cessation of pulsation in an aneurism is brought about by the deposition of fibrine in the sac, not by the coagulation of its contents, they afford us a useful practical hint in the application of pressure. For instance, after compression has been kept up for a time, if the sac becomes hard to the feel, and does not increase in size on the removal of the instrument, (although it still continues to pulsate,) we may then apply local pressure to the aneurism, by means of a compress upon the sac, with every prospect of effecting a cure more quickly than if we continued to make the pressure solely upon the artery above.—*Dub. Med. Press.*

